# Knowledge, attitudes, barriers and uptake rate of influenza virus vaccine among adults with chronic diseases in Jordan: a multicentric cross-sectional study

**DOI:** 10.3389/fpubh.2025.1603482

**Published:** 2025-06-17

**Authors:** Munir Abu-Helalah, Tarek Gharibeh, Mohammad Al-Hanaktah, Islam Khatatbeh, Fadi Batarseh, Amro Asfour, Omar Okkeh, Abdallah Dalal, Ahmad Alhendi, Huthaifa Ababneh

**Affiliations:** ^1^Department of Family and Community Medicine, Faculty of Medicine, University of Jordan, Amman, Jordan; ^2^Public Health Institute, University of Jordan, Amman, Jordan; ^3^Department of Medicine, Faculty of Medicine, University of Jordan, Amman, Jordan; ^4^Faculty of Medicine, University of Jordan, Amman, Jordan; ^5^Clinical Pharmacist, Royal Rehabilitation Center, Jordanian Royal Medical Services, Amman, Jordan; ^6^Ministry of Health, Amman, Jordan; ^7^General Surgery, Great Western Hospital, Swindon, United Kingdom; ^8^Klinikum Lüdenscheid – Germany, Orthopedics and Trauma Surgery, Lüdenscheid, Germany; ^9^Orthopedic Department, Islamic Hospital, Amman, Jordan; ^10^Internal Medicine Department, Hamad Medical Corporation, Doha, Qatar

**Keywords:** influenza vaccines, chronic diseases, uptake, barriers, attitudes, Jordan

## Abstract

**Background:**

Seasonal Influenza is a major cause of morbidity and mortality worldwide. Despite the well-established preventive role of the influenza vaccine particularly for patients at high risk, influenza vaccine uptake remains suboptimal. In Jordan, data on the influenza vaccine uptake among adults with chronic diseases at high risk of influenza complications is still lacking.

**Methods:**

A cross-sectional study was conducted to assess influenza vaccine knowledge, attitudes, uptake, and barriers among adults with selected chronic disease in Jordan. Data was collected via a structured questionnaire based on the health belief model (HBM). The face-to-face interviews were conducted between February and June 2023 at multiple healthcare centers and hospitals are representative sites of Jordan.

**Results:**

A total of 786 participants completed the study questionnaire with a mean age of 50.04 ± 15.9 years. There was a low uptake rate of influenza vaccine (10.4%) during the 2022/2023 season, while around one third of participants (32.7%) reported history of been ever vaccinated against influenza. This study identified different predictors of influenza vaccine uptake such as advice from the healthcare professional, history of complications from influenza, availability of free influenza vaccine and awareness about the vaccine availability. Worry about the vaccine safety was one of the important detected barriers.

**Conclusion:**

Similar to the global trend, particularly in developing countries, the influenza vaccine uptake rate is low among adults with different high-risk illnesses in Jordan. Results of the study provided baseline data for future interventions to improve the uptake rates of influenza vaccines in Jordan.

## Introduction

Seasonal Influenza is a major cause of morbidity and mortality worldwide as the number of severe cases reach up to 5 million cases along with half a million deaths annually (1). Furthermore, seasonal influenza epidemics significant impacts the degree of control of chronic illnesses particularly for those at a higher risk of influenza complications (2, 3). Patients with chronic cardiac and respiratory diseases, diabetic patients, patients with primary or secondary immune deficiency are at higher risk of complications from influenza infections when compared with patients free of these illness and matched for age. Influenza vaccine has been shown to reduce the morbidity and mortality for these high risk groups through decreasing complications, hospitalizations and intensive care unit admissions (4).

Despite the well-established preventive role of the influenza vaccine particularly for patients at high risk, influenza vaccine uptake remains suboptimal among adults with chronic diseases worldwide. A study from Italy attributed the low vaccination rates in this high-risk patients to knowledge gaps, misconceptions and negative attitudes toward vaccines (5). Similarly, in Korea, despite the vaccination campaigns targeting adults aged ≥50 with chronic diseases, the influenza vaccine uptake rates remain below target levels. A recent study from Jazan, Saudia Arabia revealed a low uptake of influenza vaccine uptake among 249 participants with a chronic disease. Only 103 (41.4%) ever received the influenza vaccine and only 43 (17.3%) of them received the vaccine annually (6).

HBM is a well-established behavioral model for predicting vaccines uptake and attitudes toward vaccines (7–11). Other models that have been used to assess the vaccine uptake and its predictors such as the Theory of Planner Behavior (12), Protection Motivation Theory (13), and the Social Cognitive Theory (14). Limited research has been conducted on influenza vaccine uptake among adults with chronic diseases in the Middle East and North African region where vaccination coverage rates are expected to be similarly low (15). In Jordan, the uptake rates of influenza vaccine among selected high risk groups have been studied (16, 17); however, data on the uptake rates of adults with chronic diseases at high risk of influenza complications is still lacking.

## Methodology

### Study design

A cross-sectional study was conducted to assess influenza vaccine knowledge, attitudes, uptake and barriers among adults with selected chronic diseases in Jordan. Data was collected through face-to-face interviews utilizing a structured questionnaire at multiple healthcare centers and hospitals. The study was conducted in Amman, the capital of Jordan which represents the central region; Zarqa city, central region; Irbid, northern region and Karak, southern region.

### Eligibility criteria

- The inclusion criteria was based on recruitment of Jordanian nationals, aged 18 or older, living permanently in the study area and have been diagnosed with for more than 12 months with diabetes, chronic respiratory and/or cardiovascular diseases (CVDs), rheumatological disorder on regular high dose steroid or on immune suppressants, a primary immune deficiency or suffering from a disease and leading to secondary immune deficiency. Selection was based on the WHO recommendations for chronic diseases at high risk of influenza complications (18). On the other hand, participants not living permanently in study areas or participants with contraindications to influenza vaccine were excluded from the study. Subjects were excluded in the reported that influenza vaccine is contraindicated such as history of severe allergic reaction (e.g., anaphylaxis) to any component of the vaccine (other than egg), or to a previous dose of any influenza vaccine (any egg-based IIV, ccIIV, RIV, or LAIV of any valency).

### Study sites

Participants were recruited from:

1. Al Bashir Hospitals, Amman, Jordan
2. Princess Rahma Hospital for Pediatrics, Irbid
3. Princess Basma Hospital, Irbid
4. Princess Badiea Hospital for Obstetrics and Gynecology, Irbid
5. Zarqa Governmental Hospital
6. Karak Governmental Hospital
7. Comprehensive healthcare centers from study areas: Amman comprehensive healthcare centers, New Zarqa Comprehensive Center, Karak Comprehensive Center, Irbid Comprehensive Center.

Sampling technique: A multistage sampling technique was utilized to ensure probability sampling. Sample size was distributed on the above centers according to the expected number of eligible participants at each center. Participants were recruited on different times and days including Saturday, a weekend day in Jordan, to avoid convenient sampling.

### Study tool

A structured questionnaire was specifically developed for the purpose of this study. The questionnaire was developed based on validated questionnaires that then were translated into Arabic through a backward-forward translation process conducted by public health experts (7–11). Internal consistency was assessed during the pilot phase. It provided an acceptable threshold and met the minimum cut-off of 0.60 and above. Cronbach’s Alpha ranged between 0.76 and 0.87.

The first section captured demographic data, medical and drug history, and socioeconomic factors. The second part of the questionnaire, on the other hand, covered influenza vaccine uptake and vaccine knowledge. The last part consisted of close-ended questions organized into key areas based on the Health Belief Model (HBM) addressing the perceived susceptibility, severity, benefits and barriers to influenza vaccination (7–11).

### Health belief model framework

The HBM has been used to facilitate the assessment of perceptions and attitudes of patients toward influenza vaccination (7–11). The following components of HBM were utilized in the study: the patient’s perceived risk of getting influenza (perceived susceptibility), the belief of the resulting consequences of influenza infections (perceived severity), the potential positive benefits of influenza vaccine (perceived benefits), the perceived barriers to influenza vaccine, vaccine availability, exposure to factors which prompt action (cues to action) and modifying variables (a person’s characteristics).

According to the HBM, patients’ readiness to take action (to get vaccinated) depends on the following beliefs or conditions: Their susceptibility to influenza, seriousness of the threat of influenza to their health, the benefits and risks of influenza vaccine, the benefits of taking the vaccine outweighing the risks, confidence in taking the vaccination safely and cues-to-action present to motivate vaccination.

### Questionnaire development and pilot testing

To ensure clarity, acceptability and relevance, the questionnaire was piloted on 30 patients within the study areas. These pilot interviews helped refine the questionnaire’s format and content for clarity, length and participants’ comprehension. The final version incorporated close-ended questions that were organized into key areas based on the HBM including perceived susceptibility, severity, benefits and barriers to influenza vaccination.

### Data collection

Data were collected between February and June 2023 for the 2022/2023 season vaccine uptake, noting that the vaccine is recommended in Jordan in October annually and is given until early December 2022.

A group of senior medical students and internship doctors trained on the study protocol and questionnaire assisted in recruiting the targeted representative sample from study sites through face-to-face interviews. They explained the study objectives and components of the questionnaire to eligible subjects. Upon consenting study participants, data was collected through face-to-face interview.

If the patient was unable to provide written consent, a family member signed the consent form on his/her behalf. If no one is available, an independent healthcare staff signed on behalf of the study participants. Study coordinators clearly stated that the decision about participation in the study will not affect the care given. Data collection was scheduled across varied hours and days to maximize representation of the sample.

### Sample size calculation

There are 6′415’185 adults aged above 18 years in Jordan (19). Data shows that chronic illnesses are common in Jordan with rates reaching 34% for type two diabetes (19, 20).

Based on Mullan formula, a confidence limit of 99%, a population sample of 50% and a sampling error of 5% the number of samples required has been calculated as 664. A Sample of 786 was collected to allow for comparison in attitudes, knowledge and perceptions between patients who received influenza vaccine with patients who did not (21).

### Statistical analysis

SPSS software version 28.0 was used to analyze the data. Categorical variables were summarized using descriptive statistics such as frequencies and percentages. Chi-square was performed to examine the relationship between baseline characteristics, vaccination, and level of knowledge. Binary logistic regression by backward stepwise was performed to determine the predictors of vaccination for the 2022–2023 season and predictors of lifetime influenza vaccination. The independent variables were sociodemographic factors and perceived susceptibility factors, perceived severity, perceived benefits, cues to action, and personal health factors. According to the lifetime influenza vaccination model, the Hosmer-Lemeshow test showed a good model fit (χ^2^ = 9.245, df = 8, *p* = 0.322), thereby confirming that the model was a good reflection of the data. For the vaccination model of the current season, the Hosmer-Lemeshow test indicated perfect fit (χ^2^ = 2.853, df = 8, *p* = 0.943). Robustness tests involved dropping borderline significant variables, sample splitting by gender, and stricter inclusion and exclusion criteria for variables. The findings were replicated in all of the robustness tests, where important predictors, including perceived risk to influenza, concern regarding side effects of vaccines, health professional advice, access to sufficient safety information, and immune response-affecting medical conditions (e.g., corticosteroid administration), were shown consistently to be significant. Additionally, the outcome of the Hosmer-Lemeshow test across all of the tests always revealed a proper fit for the model (all *p* > 0.05), thereby reinforcing the validity and stability of the findings.

## Results

### Demographic characteristics

A total of 786 participants were enrolled in the study, of which 54% were recruited through comprehensive primary healthcare centers and 46% through outpatients’ clinics at the selected hospitals. The mean age of study participants was 50.04 ± 15.9 years. In terms of jobs, 198 (25.2%) had full-time jobs while 54 (6.9%) had part-time jobs, 188 (23.9%) were retired and the remaining majority 346 (44.0%) were unemployed. The majority of participants lived in the city (79.1%) and the remaining 20.9% lived in a village. As far as education is concerned, 43.9% of participants have attained an educational level of high school or below while the remaining participants have had higher levels of education.

### Vaccination status with professional and demographic characteristics

Among the 786 participants who took part in the study, 82 (10.4%) received influenza vaccine during the season of 2022/2023 while around one third of participants (32.7%) reported history of ever been vaccinated against influenza. Several characteristics were substantially associated with vaccination status for the 2022/2023 season. Male participants had higher vaccination rates than female participants for both; ever (39.5% vs. 27.4%, *p* < 0.001) and during the season of data collection (13.4% vs. 8.1%, *p* = 0.017). Participants with higher educational levels also had higher rates of ever receiving the influenza vaccine (*p* < 0.001). Finally, higher family income was a significant determinant of whether participants had ever received the influenza vaccine (*p* = 0.007; Table 1).

**Table 1:** Baseline characteristics and of participants by vaccination status.

Baseline characteristics	Raw total		Vaccination status							
			Have you ever had the flu vaccine before?				Have you had the flu vaccine during this year?			
			Yes		No		Yes		No	
	N	%	N	%	N	%	N	%	N	%
Age										
<40	182	23.2	60	33.0	122	67.0	22	12.1	160	87.9
40–65	493	62.7	165	33.5	328	66.5	53	10.8	440	89.2
>65	111	14.1	32	28.8	79	71.2	7	6.3	104	93.7
	*p*-value		0.640				0.271			
	Mean Age		50.04 ± 15.9							
Gender										
Male	344	43.8	136	39.5	208	60.5	46	13.4	298	86.6
Female	442	56.2	121	27.4	321	72.6	36	8.1	406	91.9
	*p*-value		<0.001				0.017			
Residence (1)										
Amman	384	48.9	124	32.3	260	67.7	35	9.1	349	90.9
Irbid	147	18.7	52	35.4	95	64.6	16	10.9	131	89.1
Karak	172	21.9	60	34.9	112	65.1	23	13.4	149	86.6
Tafeeleh	3	0.4	0	0.0	3	100.0	0	0.0	3	100.0
Zarqa	80	10.2	21	26.3	59	73.8	8	10.0	72	90.0
	*p*-value		0.427				0.609			
Residence (2)										
City	622	79.1	203	32.6	419	67.4	62	10.0	560	90.0
Village	164	20.9	54	32.9	110	67.1	20	12.2	144	87.8
	*p*-value		0.944				0.407			
Education										
Uneducated	32	4.1	6	18.8	26	81.3	3	9.4	29	90.6
Primary school (until 10th grade)	141	18	33	23.4	108	76.6	14	9.9	127	90.1
High school (11 to 12th grades)	171	21.8	47	27.5	124	72.5	12	7.0	159	93.0
Diploma	149	19.0	43	28.9	106	71.1	12	8.1	137	91.9
BA	249	31.7	108	43.4	141	56.6	35	14.1	214	85.9
Master’s/Phd	44	5.6	20	45.5	24	54.5	6	13.6	38	86.4
	*p*-value		<0.001				0.235			
Job										
Full-time job	198	25.2	72	36.4	126	63.6	21	10.6	177	89.4
Part-time job	54	6.9	24	44.4	30	55.6	11	20.4	43	79.6
Retired	188	23.9	72	38.3	116	61.7	23	12.2	165	87.8
Unemployed	346	44.0	89	25.7	257	74.3	27	7.8	319	92.2
	*p*-value		0.002				0.030			
Family Income										
less than 500	359	45.7	95	26.5	264	73.5	32	8.9	327	91.1
500–1,000	285	36.3	105	36.8	180	63.2	28	9.8	257	90.2
1,000–2000	91	11.6	36	39.6	55	60.4	12	13.2	79	86.8
	51	6.5	21	41.2	30	58.8	10	19.6	41	80.4
	*p*-value		0.007				0.096			
Health Insurance										
No health insurance	104	13.2	32	30.8	72	69.2	3	2.9	101	97.1
MOH	430	54.7	128	29.8	302	70.2	43	10.0	387	90.0
Private	76	9.7	34	44.7	42	55.3	18	23.7	58	76.3
RMS	129	16.4	48	37.2	81	62.8	16	12.4	113	87.6
Other non-private health insurance	47	6.0	15	31.9	32	68.1	2	4.3	45	95.7
	*p*-value		0.089				<0.001			

### Medical history of participants by vaccination status

The most commonly reported disease by the study participants was cardiovascular disease (CVD; 58.3%) followed by Diabetes Mellitus (DM) which was reported by 38.2% of the participants (31.7% type II DM and 6.5% with type I DM). Rheumatological conditions on high dose corticosteroid or immune suppressants came next, it was reported by 27.7% of the study participants. Chronic Obstructive Pulmonary Disease and bronchial asthma contributed to 25.8% of the participants.

Participants with CVDs were less likely to have ever received the flu vaccine compared to those without CVDs (29.7% vs. 36.9%, *p* = 0.034). The same trend was observed for receiving the influenza vaccine during 2022/2023 season (8.1% and 13.7, respectively, *p* = 0.011). On the contrary, individuals with respiratory diseases had a significantly higher rate of ever been vaccinated compared to those with no respiratory diseases (43.3% vs. 29.0%, *p* < 0.001). However, when comparing the uptake rate for that season, these differences became small and insignificant (12.8% vs. 9.6%, *p* = 0.199). There were no statistically significant differences in the uptake of influenza vaccine with history of DM, rheumatological disorders or immune deficiencies (Table 2). Previous hospitalization due to influenza complications was significantly associated with higher vaccination rates for both ever been vaccinated (45.2% vs. 31.2%, *p* = 0.010) and for the 2022–2023 season uptake (22.6% vs. 9.0%, *p* < 0.001; Table 2).

**Table 2:** Medical history of participants by vaccination status.

Medical history	Raw total		Vaccination status							
			Have you ever had the flu vaccine before?				Have you had the flu vaccine during this year?			
			Yes		No		Yes		No	
	N	%	N	%	N	%	N	%	N	%
Do you have any cardiovascular diseases?										
Yes	458	58.3	136	29.7	322	70.3	37	8.1	421	91.9
No	328	41.7	121	36.9	207	63.1	45	13.7	283	86.3
*p*-value			0.034				0.011			
Do you have any respiratory diseases?										
Yes	203	25.8	88	43.3	115	56.7	26	12.8	177	87.2
No	583	74.2	169	29.0	414	71.0	56	9.6	527	90.4
*p*-value			<0.001				0.199			
Do you have Diabetes?										
DM Type 1	51	6.5	17	33.3	34	66.7	6	11.8	45	88.2
DM Type 2	249	31.7	75	30.1	174	69.9	20	8.0	229	92.0
No	486	61.8	165	34.0	321	66.0	56	11.5	430	88.5
*p*-value			0.575				0.325			
Do you have any rheumatological diseases taking steroids or immune suppressants regularly?										
Yes	218	27.7	75	34.4	143	65.6	22	10.1	196	89.9
No	568	72.3	182	32.0	386	68.0	60	10.6	508	89.4
*p*-value			0.528				0.846			
Do you suffer from diseases that affect immunity?										
Yes	156	19.8	47	30.1	109	69.9	17	10.9	139	89.1
No	630	80.2	210	33.3	420	66.7	65	10.3	565	89.7
*p*-value			0.445				0.823			
Do you take medications regularly?										
Yes	564	71.8	188	33.3	376	66.7	58	10.3	506	89.7
No	222	28.2	69	31.1	153	68.9	24	10.8	198	89.2
*p*-value			0.545				0.828			
Have you heard about the flu vaccine before?										
Yes	681	86.6	251	36.9	430	63.1	78	11.5	603	88.5
No	105	13.4	6	5.7	99	94.3	4	3.8	101	96.2
*p*-value			<0.001				0.017			
Do you get the flu frequently?										
Yes	172	21.9	72	41.9	100	58.1	28	16.3	144	83.7
No	614	78.1	185	30.1	429	69.9	54	8.8	560	91.2
*p*-value			0.004				0.005			
Have you been hospitalized previously due to flu complications?										
Yes	84	10.7	38	45.2	46	54.8	19	22.6	65	77.4
No	702	89.3	219	31.2	483	68.8	63	9.0	639	91.0
*p*-value			0.010				<0.001			

### Perceived susceptibility and severity of influenza and vaccination status

Table 3 shows the effect of perceived susceptibility and severity of influenza on vaccination status. The perception of increased susceptibility and severity was associated with increased vaccination rates. When compared with other participants, people who felt they are more susceptible to catching the flu compared to other people had higher vaccination rates during both 2022–2023 season (17.7% vs. 7.3%, *p* < 0.001) and for reporting ever been vaccinated (44.6% vs. 27.2%, respectively, *p* < 0.001). The same trend was also seen with those who felt at risk without the influenza vaccine (*p* < 0.001) and for those who felt that getting the flu puts them at increased risk of serious complications (*p* < 0.001).

**Table 3:** The association between perceived susceptibility and severity of influenza and vaccination status.

Perceived susceptibility and severity of influenza	Vaccination status									
	Raw total		Have you ever had the flu vaccine before?				Have you had the flu vaccine during this year?			
			Yes		No		Yes		No	
	N	%	N	%	N	%	N	%	N	%
Do you catch the flu more easily than other people of the same age?										
Strongly Agree, Agree	231	29.4	103	44.6	128	55.4	41	17.7	190	82.3
Neutral	184	23.4	53	28.8	131	71.2	14	7.6	170	92.4
Strongly Disagree, Disagree	371	47.2	101	27.2	270	72.8	27	7.3	344	92.7
*p-*value			<0.001				<0.001			
Without the vaccine, I feel like I’m at high risk of getting the flu										
Strongly Agree, Agree	262	33.3	131	50.0	131	50.0	60	22.9	202	77.1
Neutral	229	29.1	69	30.1	160	69.9	12	5.2	217	94.8
Strongly Disagree, Disagree	295	37.5	57	19.3	238	80.7	10	3.4	285	96.6
*p*-value			<0.001				<0.001			
If I get the flu, I get severe symptoms										
Strongly Agree, Agree	341	43.4	140	41.1	201	58.9	45	13.2	296	86.8
Neutral	164	20.9	47	28.7	117	71.3	15	9.1	149	90.9
Strongly Disagree, Disagree	281	35.8	70	24.9	211	75.1	22	7.8	259	92.2
*p*-value			<0.001				0.077			
If I get the flu, I get moderate symptoms										
Strongly Agree, Agree	501	63.7	170	33.9	331	66.1	56	11.2	445	88.8
Neutral	172	21.9	50	29.1	122	70.9	16	9.3	156	90.7
Strongly Disagree, Disagree	113	14.4	37	32.7	76	67.3	10	8.8	103	91.2
*p*-value			0.503				0.658			
If I get the flu, I get myself at risk of serious complications										
Strongly Agree, Agree	240	30.5	108	45.0	132	55.0	42	17.5	198	82.5
Neutral	158	20.1	48	30.4	110	69.6	11	7.0	147	93.0
Strongly Disagree, Disagree	338	43.0	101	26.0	287	74.0	29	7.5	359	92.5
*p*-value			<0.001				<0.001			
If I get the flu, I put those around me at risk of infection										
Strongly Agree, Agree	579	73.7	211	36.4	368	63.6	68	11.7	511	88.3
Neutral	105	13.4	20	19.0	85	81.0	5	4.8	100	95.2
Strongly Disagree, Disagree	102	13.0	26	25.5	76	74.5	9	8.8	93	91.2
*p*-value			0.001				0.084			

### Perceived benefits vs. barriers to influenza vaccination

When evaluating the association between perceived benefits and vaccination status (Table 4), several factors significantly influenced vaccination rates. Participants who reported a reduced worry about contracting influenza if vaccinated believed in the vaccine’s benefits and those who recognized its role in reducing the spread of infection had statistically significant higher lifetime influenza vaccination rates and higher influenza vaccine uptake during the season of 2022–2023 (*p* < 0.05 for all variables).

**Table 4:** The association between perceived benefits and vaccination status.

Perceived benefit of vaccination	Raw total		Vaccination status							
			Have you ever had the flu vaccine before?				Have you had the flu vaccine during this year?			
			Yes		No		Yes		No	
	N	%	N	%	N	%	N	%	N	%
I will not worry about getting the flu if I get the flu vaccine										
Strongly Agree, Agree	350	44.5	144	41.1	206	58.9	50	14.3	300	85.7
Neutral	225	28.6	57	25.3	168	74.7	15	6.7	210	93.3
Strongly Disagree, Disagree	211	26.8	56	26.5	155	73.5	17	8.1	194	91.9
*p*-value			<0.001				0.006			
Getting the flu vaccine will benefit me										
Strongly Agree, Agree	503	64.0	209	41.6	294	58.4	71	14.1	432	85.9
Neutral	192	24.4	34	17.7	158	82.3	8	4.2	184	95.8
Strongly Disagree, Disagree	91	11.6	14	15.4	77	84.6	3	3.3	88	96.7
*p*-value			<0.001				<0.001			
Getting the flu vaccine will reduce the percentage of people around me infected with the flu?										
Strongly Agree, Agree	510	64.9	209	41.0	301	59.0	71	13.9	439	86.1
Neutral	165	21.0	22	13.3	143	86.7	5	3.0	160	97.0
Strongly Disagree, Disagree	111	14.1	26	23.4	85	76.6	6	5.4	105	94.6
*p*-value			<0.001				<0.001			

On the other hand, a range of perceived barriers was associated with lower vaccination rates with varying levels of significance. These barriers include concerns about side effects, a general dislike of vaccines, the belief that the vaccine could cause influenza and apprehension regarding unknown aspects of the vaccine. Additional barriers such as knowing someone who had a negative experience with the vaccine, lack of encouragement from the family, friends, or coworkers, difficulty scheduling appointments, time constraints, vaccine cost, lack of insurance coverage, limited availability, the belief that the vaccine is unsafe and fear or dislike of needles have been included (Supplementary Table 1).

Among the unvaccinated participants, the most commonly endorsed barrier to vaccination was concern about insufficient knowledge on the flu vaccine (*n* = 318, *p* < 0.001) followed by a general dislike of vaccinations (*n* = 296, *p* < 0.001). Interestingly, the statement most frequently disagreed with by unvaccinated participants was, “Someone I know had a bad experience with the flu vaccine” (*n* = 343, *p* = 0.005; Supplementary Table 1).

### The association between perceived cues to action and vaccination status

Supplementary Table 2 highlights the association between cues to action and vaccination status. A recommendation from a healthcare professional was the most influential factor with 76.5% of participants (*n* = 601) agreeing that it is important for making the right decision regarding the vaccine. This was significantly associated with both having ever been vaccinated (*p* < 0.001) and been vaccinated during the data collection season (*p* = 0.003).

Additionally, trust in vaccination guidelines and the desire for comprehensive information about the vaccine were significant factors influencing vaccination rates (*p* < 0.001). While the availability of the vaccine free of charge was also significant (*p* < 0.001) where half of the participants agreed that it was an encouraging factor (Supplementary Table 2).

### Effect of information and its sources on vaccination rates

As shown in Supplementary Table 3, any source of information had a significant effect on vaccination rates among participants (*p* < 0.001). Interestingly, when asked about various sources of information regarding the influenza vaccine, friends, relatives, and colleagues were the most frequently cited with 55.1% (*n* = 434) of participants reporting “yes.” This was followed by doctors where 48.0% (*n* = 377) reported that they received information from this source.

Finally, about two thirds of participants (63.2%, *n* = 497) reported feeling that they did not have enough information about the safety and side effects of the flu vaccine (*p* < 0.001; Supplementary Table 3).

### Factors affecting the probability of participants getting the influenza vaccine

The logistic regression analysis in Table 5A identified key predictors for receiving the influenza vaccine during the 2022–2023 season. Having private health insurance significantly increased the likelihood of vaccination (*p* = 0.037). Perceived risk without the vaccine more than doubled vaccination rates (*p* < 0.001), conversely, worry about side effects almost halved vaccination rates (*p* < 0.001). Time constraints were another significant barrier; participants who reported being too busy have decreased odds of vaccination (OR = 0.615, *p* = 0.011). Advice from doctors had a strong positive influence of more than doubling the vaccination rates (OR = 2.587, *p* = 0.003). Finally, having adequate information about the safety and side effects of the vaccine was the most impactful factor; more than tripling the odds of vaccination (OR = 3.016, *p* < 0.001; Table 5A).

**Table 5:** Predictors of influenza vaccine uptake based on the logistic regression analysis.

Predictor	B	*p*-value	Odds ratio	95% C.I	
				Lower	Upper
A. During the season of 2022/2023					
Health insurance (Private)-Cues to actions	0.203	0.037	1.225	1.013	1.482
Without the vaccine, I feel like I’m at high risk of getting the flu-Perceived susceptibility	0.977	<0.001	2.657	1.805	3.909
If I get the flu, I get myself at risk of serious complications-Perceived severity	0.293	0.068	1.340	0.979	1.836
Worry about side effects-Perceived Barriers	−0.555	<0.001	0.574	0.421	0.784
I do not have time to get the flu vaccine-Perceived Barriers	−0.486	0.011	0.615	0.422	0.896
Advice from a doctor-Cues to action	0.950	0.003	2.587	1.378	4.857
Enough information about the safety and side effects of the vaccine?-Cues to action	1.104	<0.001	3.016	1.677	5.425
Taking corticosteroids regularly-Perceived susceptibility	0.767	0.050	2.154	0.999	4.646
B. Predictors of ever receiving influenza vaccine					
Without the vaccine, I feel like I’m at high risk of getting the flu-Perceived susceptibility	0.411	0.001	1.508	1.185	1.918
If I get the flu, I get myself at risk of serious complications Perceived severity	0.339	0.004	1.403	1.114	1.766
I am worried about the side effects of the flu vaccine-Perceived Barriers	−0.427	<0.001	0.653	0.517	0.824
Someone I know had a bad experience with the flu vaccine-Perceived Barriers	0.351	0.012	1.421	1.080	1.869
I am afraid/do not like needles, so I do not get the flu vaccine-Perceived Barriers	−0.526	<0.001	0.591	0.455	0.768
I do not have time to get the flu vaccine-Perceived Barries	−0.322	0.012	0.725	0.565	0.931
I trust the guidelines that recommend that all high-risk groups should get the flu vaccine. Self-efficacy	0.303	0.069	1.355	0.976	1.880
I feel I have received all the information I need to decide if I should get the flu vaccine-Cues to actions	0.287	0.033	1.332	1.024	1.732
Have you ever received advice from a doctor about taking the flu vaccine? Cues to action	0.514	0.019	1.672	1.087	2.572
Have you ever received advice from a pharmacist about taking the flu vaccine? Cues to action	0.789	<0.001	2.200	1.442	3.357
Has anyone or a source influenced you not to get the flu vaccine? Perceived Barriers	−0.527	0.040	0.591	0.357	0.977
Do you have enough information about the safety and side effects of the flu vaccine? Cues to action	0.558	0.012	1.747	1.132	2.696
Do you take medications regularly? Perceived Susceptibility	0.381	0.082	1.463	0.953	2.247
Have you heard about the flu vaccine before? Cues to action	1.828	<0.001	6.222	2.479	15.615

The pseudo R^2^ values for the final logistic regression model are as follows: Cox and Snell R^2^ = 0.207, Nagelkerke R^2^ = 0.425. The pseudo R^2^ values for the final logistic regression model are as follows: Cox and Snell R^2^ = 0.340, Nagelkerke R^2^ = 0.474. These results suggest a moderate level of explanatory power for the model.

Regression analysis was repeated for predictors of “having ever received the influenza vaccine,” as shown in Supplementary Table 5B. Perceived risk without the vaccine increased the odds of vaccination by 1.508 times (*p* = 0.001), similarly, perceiving a risk of serious complications from the flu raised the likelihood of vaccination by 1.403 times (*p* = 0.004).

Concerns about vaccine side effects significantly reduced the odds of vaccination (OR = 0.653, *p* < 0.001), as did the fear of needles (OR = 0.591, *p* < 0.001) and the lack of time to get vaccinated (OR = 0.725, *p* = 0.012; Table 5B).

Receiving adequate information about the vaccine significantly increased the vaccination rates (OR = 1.332, *p* = 0.033). Advice from a doctor (OR = 1.67, *p* = 0.019) or a pharmacist (OR = 2.20, *p* < 0.001) also strongly predicted vaccine uptake. Having sufficient information about the vaccine’s safety and side effects was also another key predictor (OR = 1.747, *p* = 0.012). Hearing about the flu vaccine before the study was the strongest factor to increase vaccination odds by more than sixfold (OR = 6.222, *p* < 0.001; Table 5B).

## Discussion

This study showed that the influenza vaccine uptake was significantly low in Jordan among adult patients at high risk of influenza complications. The reported rates of 10.4% during 2022–2023 season and 32.7% reported history of ever been vaccinated against influenza is aligned with the reported global trend of low vaccination rate among patients with chronic disease (5, 6, 16, 22–24). In one Jordanian study that survey older adults subjects above 65 years old, data showed a vaccination rate of only 1.2% (17). Gender disparity in vaccine uptake was observed where males were more likely to get vaccinated. A similar report from Saudi Arabia indicated that being a male increased the odds of vaccination by 73% (OR 1.73) (25). This reproducible finding can be explained by the fact that higher vaccine side effects occur in females (26, 27).

Similar to previous studies, socioeconomic factors are important predictors of the influenza vaccine uptake. Higher level of education and income and residence in urban area were among the factors that predict higher vaccination rates (28, 29). These findings underscore the importance of implementing organized strategies to improve vaccination coverage among low-income and rural populations.

Understandably, patients who were previously hospitalized because of influenza have a higher vaccination rate. A global assessment of the predictors of influenza vaccine uptake through a meta-analysis of 522 studies from 68 countries/region revealed that the perceived risk is one of the important predictors of influenza vaccine uptake, along with some disparities in the uptake rates between developed and developing countries. A free national or regional vaccination policy, perception of influenza vaccine efficacy and disease severity, a recommendation from healthcare workers and having a history of influenza vaccination were positive predictors of vaccine uptake (*p* < 0.01) (29).

A high complication rate from influenza infections has been reported in patients with cardiovascular disease (CVD) which is among the most common conditions observed in adults hospitalized due to influenza (30). Despite this, influenza vaccination rates remain low in this population group. Vaccination coverage varies significantly; for example, the PARADIGM-HF trial reported a vaccination rate of 21% in patients with heart failure, (31) while another study observed a 45% rate among patients with ischemic heart disease (32). In our survey, 29.7% of patients with cardiovascular disease reported receiving an influenza vaccine.

For patients with respiratory diseases, 43% reported receiving vaccination during the season surveyed indicating a rate higher than the previously reported (33–35) but still falls below the target recommended by the World Health Organization (WHO). Similar barriers to receiving the influenza vaccine have been reported in patients with respiratory diseases like COPD; lack of knowledge, misperception about vaccine effectiveness are common contributors (36, 37).

In diabetic patients, vaccination rate varies widely; studies reported rates between 28 and 61% (38, 39). In our survey, the vaccination rate in this group was 33%, aligning with the lower end of the globally reported range. The wide variation in vaccination rates among patients with chronic illnesses observed across studies is likely attributed to cultural differences, education levels and the presence/absence of vaccination programs in these populations.

Vaccination hesitancy is common across all vaccines, particularly for influenza vaccination, as it needs to be administered annually, has variable effectiveness and is associated with a high rate of self-reported side effects (37, 40) This issues has increased globally post COVID-19 pandemic. Vaccination hesitancy has been considered by the World Health Organization as a danger to global health (41). A false belief that individuals are less likely to contract influenza or develop complications was reported in an Australian study. Another myth that influenza vaccine can cause serious influenza infection has been reported. In this study, the two most stated reasons for refusing the vaccine were ‘the situation is not serious enough’ and ‘I am not at risk’ (42). In contrary, subjects who are worried about getting infected, have higher rates of influenza vaccination (43). Therefore, perceived susceptibility of being at high risk is an important predictor of influenza vaccine uptake, therefore, healthcare professionals and future health promotion activities can target this important component of the health belief model to improve influenza vaccine uptake.

Our survey identified several barriers to influenza vaccine uptake including concerns about side effects, the general dislike of vaccines, the belief that the vaccine could cause influenza and apprehension about unknown aspects of the vaccine. These findings are consistent with several previous reports (44–46). Having a negative attitude toward the influenza vaccine was a major barrier to vaccine uptake which has been reported in a large study where older adult participant expressed negative thoughts above the vaccination (47). A belief that one could still get influenza after being vaccinated and the fear of side effects have been reported in 92.6 and 29.5% of subjects, respectively, in a Qatari study (48). Also, lack of social pressure from friends and family was identified as a predictor of low vaccination uptake as lower vaccination rate among older adult people who live alone was noted in one study (49). An important cues to action to improve the uptake of the influenza vaccine is improving knowledge about adverse drug reactions for the target high risk groups.

This survey emphasizes the role of healthcare providers in increasing the vaccination uptake rate. Their influential role is of paramount importance. Similar other reports identified this rule; less interaction with health care like low rate of physician interactions and lack of a primary care physician were associated with low rate of vaccine uptake (50). Knowledge and education provided by healthcare professionals play a crucial role in alleviating patient concerns (such as the ones related to efficacy and side effects) and debunking faulty perceptions about the influenza vaccine. Consequently, patients interaction with an informed healthcare provider increases the likelihood of receiving the vaccine, and this has been identified as one of the key cues to action to improve influenza vaccine uptake (51–53).

Despite the benefits demonstrated, influenza vaccination has been historically underutilized in both the general adult population and patients with chronic illnesses. In a study of patients with atherosclerotic cardiovascular disease, there was a low uptake rate of 37% of those aged 18–49 years and 55% for those aged 50–64 years in 2019–2020 (54). It was recommend that annual influenza vaccination should be administered, together with other guideline-recommended therapies aimed at reducing cardiovascular risk, to patients with a cardiovascular indication (54, 55). The same approach is needed for different high-risk patients.

Although, our study is one of the few from the Middle East that target patients with chronic diseases and are at high risk of influenza infections, it has several limitations. One of the key limitations is that the study was that it was based on self-reported influenza vaccine uptake without confirmation from medical records. The same is applied for not confirming contraindications from medical notes. In Jordan, influenza vaccine is provided by physicians at clinics and hospitals and by community pharmacists. This has limited the confirmation of the uptake. The study included several conditions and did not focus on specific diseases. It is recommended that future studies should be disease specific to understand the needs for different groups in more detail. Moreover, campaigns targeting specific patient-groups would be of more value particularly focusing on negative outcomes of influenza infections for these patients (56). Finally, this study is representative of the public sector in Jordan, therefore, it is recommended to conduct a study to include the private sector in Jordan.

## Conclusion

In conclusion, the influenza vaccine uptake is low among adult with different high-risk illnesses. This study identified several psychological, physical, sociodemographic and contextual barriers that contributed to low influenza vaccination uptake. The study also emphasized on the important role of healthcare providers and existing guidelines implementation in improving the influenza vaccine uptake for patients at high risk of influenza complication. Improvement of knowledge about the perceived risk of influenza and the safety of the vaccine could play an important role in improving influenza vaccine uptake in Jordan. This in addition to providing comprehensive data to assist future interventions to improve the reported low uptake rates in Jordan.

## Data Availability

The original contributions presented in the study are included in the article/Supplementary material, further inquiries can be directed to the corresponding author.
