# Supplementary material for: Knowledge, attitudes, barriers and uptake rate of influenza virus vaccine among adults with chronic diseases in Jordan: a multicentric cross-sectional study
Source: Front Public Health. 2025 Jun 17;13:1603482. doi: 10.3389/fpubh.2025.1603482 (PMC12209355; doi:10.3389/fpubh.2025.1603482)
Supplement: Supplementary file 1 [file Supplementary_file_1.docx]

| **Supplementary table 1: The association between Perceived barriers and vaccination status.** | | | | | | | | | | | | | | | | | | | |
| --- | --- | --- | --- | --- | --- | --- | --- | --- | --- | --- | --- | --- | --- | --- | --- | --- | --- | --- | --- |
|  |  | |  | |  |  | |  | |  | |  |  |  | |  | |  | |
|  | | | **Raw total** | | | **Have you ever had the flu vaccine before?** | | | | | | | **Have you had the flu vaccine during this year?** | | | | | | |
|  |  |  |  |  |  | **YES** | | | | **NO** | | | **YES** | | | **NO** | | | |
|  |  |  | N | | % | N | | % | | N | | % | N | % | | N | | % | |
| **I am worried about the side effects of the flu vaccine** | **Strongly Agree, Agree** | | 365 | | 46.4% | 91 | | 24.9% | | 274 | | 75.1% | 25 | 6.8% | | 340 | | 93.2% | |
|  | **Neutral** | | 176 | | 22.4% | 47 | | 26.7% | | 129 | | 73.3% | 10 | 5.7% | | 166 | | 94.3% | |
|  | **Strongly Disagree, Disagree** | | 245 | | 31.2% | 119 | | 48.6% | | 126 | | 51.4% | 47 | 19.2% | | 198 | | 80.8% | |
|  | ***P-value*** | | | | | ***<0.001*** | | | | | | | ***<0.001*** | | | | | | |
| **I do not like vaccinations** | **Strongly Agree, Agree** | | 374 | | 47.6% | 78 | | 20.9% | | 296 | | 79.1% | 18 | 4.8% | | 356 | | 95.2% | |
|  | **Neutral** | | 153 | | 19.5% | 58 | | 37.9% | | 95 | | 62.1% | 18 | 11.8% | | 135 | | 88.2% | |
|  | **Strongly Disagree, Disagree** | | 259 | | 33.0% | 121 | | 46.7% | | 138 | | 53.3% | 46 | 17.8% | | 213 | | 82.2% | |
|  | ***P-value*** | | | | | ***<0.001*** | | | | | | | ***<0.001*** | | | | | | |
| **The flu vaccine might give me the flu** | **Strongly Agree, Agree** | | 319 | | 40.6% | 100 | | 31.3% | | 219 | | 68.7% | 30 | 9.4% | | 289 | | 90.6% | |
|  | **Neutral** | | 223 | | 28.4% | 62 | | 27.8% | | 161 | | 72.2% | 20 | 9.0% | | 203 | | 91.0% | |
|  | **Strongly Disagree, Disagree** | | 244 | | 31.0% | 95 | | 38.9% | | 149 | | 61.1% | 32 | 13.1% | | 212 | | 86.9% | |
|  | ***P-value*** | | | | | ***0.03*** | | | | | | | 0.253 | | | | | | |
| **I am worried that there may be something I do not know about the flu vaccine** | **Strongly Agree, Agree** | | 407 | | 51.8% | 89 | | 21.9% | | 318 | | 78.1% | 24 | 5.9% | | 383 | | 94.1% | |
|  | **Neutral** | | 161 | | 20.5% | 56 | | 34.8% | | 105 | | 65.2% | 16 | 9.9% | | 145 | | 90.1% | |
|  | **Strongly Disagree, Disagree** | | 218 | | 27.7% | 112 | | 51.4% | | 106 | | 48.6% | 42 | 19.3% | | 176 | | 80.7% | |
|  | ***P-value*** | | | | | ***<0.001*** | | | | | | | ***<0.001*** | | | | | | |
| **Someone I know had a bad experience with the flu vaccine** | **Strongly Agree, Agree** | | 143 | | 18.2% | 60 | | 42.0% | | 83 | | 58.0% | 20 | 14.0% | | 123 | | 86.0% | |
|  | **Neutral** | | 135 | | 17.2% | 32 | | 23.7% | | 103 | | 76.3% | 10 | 7.4% | | 125 | | 92.6% | |
|  | **Strongly Disagree, Disagree** | | 508 | | 64.6% | 165 | | 32.5% | | 343 | | 67.5% | 52 | 10.2% | | 456 | | 89.8% | |
|  | ***P-value*** | | | | | ***0.005*** | | | | | | | 0.194 | | | | | | |
| **I was not encouraged by family members, friends, or co-workers** | **Strongly Agree, Agree** | | 190 | | 24.2% | 57 | | 30.0% | | 133 | | 70.0% | 17 | 8.9% | | 173 | | 91.1% | |
|  | **Neutral** | | 181 | | 23.0% | 38 | | 21.0% | | 143 | | 79.0% | 11 | 6.1% | | 170 | | 93.9% | |
|  | **Strongly Disagree, Disagree** | | 415 | | 52.8% | 162 | | 39.0% | | 253 | | 61.0% | 54 | 13.0% | | 361 | | 87.0% | |
|  | ***P-value*** | | | | | ***<0.001*** | | | | | | | ***0.029*** | | | | | | |
| **I am afraid/do not like needles, so I do not get the flu vaccine** | **Strongly Agree, Agree** | | 181 | | 23.0% | 34 | | 18.8% | | 147 | | 81.2% | 13 | 7.2% | | 168 | | 92.8% | |
|  | **Neutral** | | 134 | | 17.0% | 27 | | 20.1% | | 107 | | 79.9% | 9 | 6.7% | | 125 | | 93.3% | |
|  | **Strongly Disagree, Disagree** | | 471 | | 59.9% | 196 | | 41.6% | | 275 | | 58.4% | 60 | 12.7% | | 411 | | 87.3% | |
|  | ***P-value*** | | | | | ***<0.001*** | | | | | | | ***0.035*** | | | | | | |
| **It is difficult to arrange an appointment or to go to the clinic** | **Strongly Agree, Agree** | | 208 | | 26.5% | 59 | | 28.4% | | 149 | | 71.6% | 17 | 8.2% | | 191 | | 91.8% | |
|  | **Neutral** | | 146 | | 18.6% | 33 | | 22.6% | | 113 | | 77.4% | 8 | 5.5% | | 138 | | 94.5% | |
|  | **Strongly Disagree, Disagree** | | 432 | | 55.0% | 165 | | 38.2% | | 267 | | 61.8% | 57 | 13.2% | | 375 | | 86.8% | |
|  | ***P-value*** | | | | | ***0.001*** | | | | | | | ***0.014*** | | | | | | |
| **I do not have time to get the flu vaccine** | **Strongly Agree, Agree** | | 146 | | 18.6% | 39 | | 26.7% | | 107 | | 73.3% | 11 | 7.5% | | 135 | | 92.5% | |
|  | **Neutral** | | 140 | | 17.8% | 35 | | 25.0% | | 105 | | 75.0% | 7 | 5.0% | | 133 | | 95.0% | |
|  | **Strongly Disagree, Disagree** | | 500 | | 63.6% | 183 | | 36.6% | | 317 | | 63.4% | 64 | 12.8% | | 436 | | 87.2% | |
|  | ***P-value*** | | | | | ***0.008*** | | | | | | | ***0.013*** | | | | | | |
| **The flu vaccine is expensive** | **Strongly Agree, Agree** | | 230 | | 29.3% | 79 | | 34.3% | | 151 | | 65.7% | 26 | 11.3% | | 204 | | 88.7% | |
|  | **Neutral** | | 311 | | 39.6% | 76 | | 24.4% | | 235 | | 75.6% | 23 | 7.4% | | 288 | | 92.6% | |
|  | **Strongly Disagree, Disagree** | | 245 | | 31.2% | 102 | | 41.6% | | 143 | | 58.4% | 33 | 13.5% | | 212 | | 86.5% | |
|  | ***P-value*** | | | | | ***<0.001*** | | | | | | | 0.059 | | | | | | |
| **The flu vaccine is not covered in the health insurance** | **Strongly Agree, Agree** | | 343 | | 43.6% | 119 | | 34.7% | | 224 | | 65.3% | 33 | 9.6% | | 310 | | 90.4% | |
|  | **Neutral** | | 279 | | 35.5% | 64 | | 22.9% | | 215 | | 77.1% | 22 | 7.9% | | 257 | | 92.1% | |
|  | **Strongly Disagree, Disagree** | | 164 | | 20.9% | 74 | | 45.1% | | 90 | | 54.9% | 27 | 16.5% | | 137 | | 83.5% | |
|  | ***P-value*** | | | | | ***<0.001*** | | | | | | | ***0.014*** | | | | | | |
| **Flu vaccine is not available** | **Strongly Agree, Agree** | | 146 | | 18.6% | 58 | | 39.7% | | 88 | | 60.3% | 17 | 11.6% | | 129 | | 88.4% | |
|  | **Neutral** | | 283 | | 36.0% | 58 | | 20.5% | | 225 | | 79.5% | 19 | 6.7% | | 264 | | 93.3% | |
|  | **Strongly Disagree, Disagree** | | 357 | | 45.4% | 141 | | 39.5% | | 216 | | 60.5% | 46 | 12.9% | | 311 | | 87.1% | |
|  | ***P-value*** | | | | | ***<0.001*** | | | | | | | ***0.035*** | | | | | | |
| **Flu vaccine might be dangerous for patients** | **Strongly Agree, Agree** | | 202 | | 25.7% | 39 | | 19.3% | | 163 | | 80.7% | 12 | 5.9% | | 190 | | 94.1% | |
|  | **Neutral** | | 299 | | 38.0% | 86 | | 28.8% | | 213 | | 71.2% | 20 | 6.7% | | 279 | | 93.3% | |
|  | **Strongly Disagree, Disagree** | | 285 | | 36.3% | 132 | | 46.3% | | 153 | | 53.7% | 50 | 17.5% | | 235 | | 82.5% | |
|  | ***P-value*** | | | | | ***<0.001*** | | | | | | | ***<0.001*** | | | | | | |
| **I am against taking flu vaccine** | **Strongly agree, agree** | 144 | | 18.3% | | 24 | 16.7% | | 120 | | 83.3% | | 12 | | 8.3% | | 132 | | 91.7% |
|  | **Neutral** | 210 | | 26.7% | | 40 | 19.0% | | 170 | | 81.0% | | 9 | | 4.3% | | 201 | | 95.7% |
|  | **Strongly disagree, disagree** | 432 | | 55.0% | | 193 | 44.7% | | 239 | | 55.3% | | 61 | | 14.1% | | 371 | | 85.9% |
|  | ***P-value*** | | | | | ***<0.001*** | | | | | | | ***<0.001*** | | | | | | |
|  |  | |  | |  |  | |  | |  | |  |  |  | |  | |  | |
|  |  | |  | |  |  | |  | |  | |  |  |  | |  | |  | |
| **Supplementary Table 2:** **The association between Perceived cues to action and vaccination status.** | | | | | | | | | | | | | | | | | | | |
|  |  | |  | |  |  | |  | |  | |  |  |  | |  | |  | |
|  | | | **Raw total** | | | **Have you ever had the flu vaccine before?** | | | | | | | **Have you had the flu vaccine during this year?** | | | | | | |
|  |  |  |  |  |  | **YES** | | | | **NO** | | | **YES** | | | **NO** | | | |
|  |  |  | N | | % | N | | % | | N | | % | N | % | | N | | % | |
| **Recommendation of my doctor/ nurse/ pharmacist is important for making the right decision to take the vaccine** | Strongly agree, agree | | 601 | | 76.5% | 219 | | 36.4% | | 382 | | 63.6% | 75 | 12.5% | | 526 | | 87.5% | |
|  | Neutral | | 103 | | 13.1% | 22 | | 21.4% | | 81 | | 78.6% | 4 | 3.9% | | 99 | | 96.1% | |
|  | Strongly disagree, disagree | | 82 | | 10.4% | 16 | | 19.5% | | 66 | | 80.5% | 3 | 3.7% | | 79 | | 96.3% | |
|  | ***P-value*** | | | | | ***<0.001*** | | | | | | | ***0.003*** | | | | | | |
| **I trust the guidelines that recommend that all high-risk groups should get the flu vaccine.** | Strongly agree, agree | | 544 | | 69.2% | 212 | | 39.0% | | 332 | | 61.0% | 74 | 13.6% | | 470 | | 86.4% | |
|  | Neutral | | 149 | | 19.0% | 29 | | 19.5% | | 120 | | 80.5% | 5 | 3.4% | | 144 | | 96.6% | |
|  | Strongly disagree, disagree | | 93 | | 11.8% | 16 | | 17.2% | | 77 | | 82.8% | 3 | 3.2% | | 90 | | 96.8% | |
|  | ***P-value*** | | | | | ***<0.001*** | | | | | | | ***<0.001*** | | | | | | |
| **I feel I have received all the information I need to decide if I should get the flu vaccine** | Strongly agree, agree | | 443 | | 56.4% | 195 | | 44.0% | | 248 | | 56.0% | 66 | 14.9% | | 377 | | 85.1% | |
|  | Neutral | | 147 | | 18.7% | 28 | | 19.0% | | 119 | | 81.0% | 6 | 4.1% | | 141 | | 95.9% | |
|  | Strongly disagree, disagree | | 196 | | 24.9% | 34 | | 17.3% | | 162 | | 82.7% | 10 | 5.1% | | 186 | | 94.9% | |
|  | ***P-value*** | | | | | ***<0.001*** | | | | | | | ***<0.001*** | | | | | | |
| **If the flu vaccine is available for free, I'll take it** | strongly agree, agree | | 385 | | 49.0% | 179 | | 46.5% | | 206 | | 53.5% | 58 | 15.1% | | 327 | | 84.9% | |
|  | Neutral | | 221 | | 28.1% | 54 | | 24.4% | | 167 | | 75.6% | 17 | 7.7% | | 204 | | 92.3% | |
|  | Strongly disagree, disagree | | 180 | | 22.9% | 24 | | 13.3% | | 156 | | 86.7% | 7 | 3.9% | | 173 | | 96.1% | |
|  | ***P-value*** | | | | | ***<0.001*** | | | | | | | ***<0.001*** | | | | | | |
|  |  | |  | |  |  | |  | |  | |  |  |  | |  | |  | |
|  |  | |  | |  |  | |  | |  | |  |  |  | |  | |  | |
|  |  | |  | |  |  | |  | |  | |  |  |  | |  | |  | |
| **Supplementary Table 3: Participants' responses to other factors that may predict vaccination rate.** | | | | | | | | | | | | | | | | | | | |
|  |  | |  | |  |  | |  | |  | |  |  |  | |  | |  | |
|  | | | **Raw total** | | | **Have you ever had the flu vaccine before?** | | | | | | | **Have you had the flu vaccine during this year?** | | | | | | |
|  |  |  |  |  |  | YES | | | | NO | | | YES | | | NO | | | |
|  |  |  | N | | % | N | | % | | N | | % | N | % | | N | | % | |
| **Have you ever received advice from a doctor about taking the flu vaccine?** | Yes | | 377 | | 48.0% | 183 | | 48.5% | | 194 | | 51.5% | 66 | 17.5% | | 311 | | 82.5% | |
|  | No | | 409 | | 52.0% | 74 | | 18.1% | | 335 | | 81.9% | 16 | 3.9% | | 393 | | 96.1% | |
|  | ***P-value*** | | | | | ***<0.001*** | | | | | | | ***<0.001*** | | | | | | |
| **Have you ever received advice from a nurse about taking the flu vaccine?** | Yes | | 194 | | 24.7% | 106 | | 54.6% | | 88 | | 45.4% | 44 | 22.7% | | 150 | | 77.3% | |
|  | No | | 592 | | 75.3% | 151 | | 25.5% | | 441 | | 74.5% | 38 | 6.4% | | 554 | | 93.6% | |
|  | ***P-value*** | | | | | ***<0.001*** | | | | | | | ***<0.001*** | | | | | | |
| **Have you ever received advice from a pharmacist about taking the flu vaccine?** | Yes | | 275 | | 35.0% | 148 | | 53.8% | | 127 | | 46.2% | 51 | 18.5% | | 224 | | 81.5% | |
|  | No | | 511 | | 65.0% | 109 | | 21.3% | | 402 | | 78.7% | 31 | 6.1% | | 480 | | 93.9% | |
|  | ***P-value*** | | | | | ***<0.001*** | | | | | | | ***<0.001*** | | | | | | |
| **Have you received advice from anyone (****friend,relative,colleague) about taking the flu vaccine?** | Yes | | 434 | | 55.2% | 190 | | 43.8% | | 244 | | 56.2% | 66 | 15.2% | | 368 | | 84.8% | |
|  | No | | 352 | | 44.8% | 67 | | 19.0% | | 285 | | 81.0% | 16 | 4.5% | | 336 | | 95.5% | |
|  | ***P-value*** | | | | | ***<0.001*** | | | | | | | ***<0.001*** | | | | | | |
| **Has anyone or a source influenced you not to get the flu vaccine?** | Yes | | 154 | | 19.6% | 49 | | 31.8% | | 105 | | 68.2% | 21 | 13.6% | | 133 | | 86.4% | |
|  | No | | 632 | | 80.4% | 208 | | 32.9% | | 424 | | 67.1% | 61 | 9.7% | | 571 | | 90.3% | |
|  | ***P-value*** | | | | | 0.795 | | | | | | | 0.147 | | | | | | |
| **Do you know of anyone who has had severe side effects (life-threatening such as liver/kidney failure/arrhythmia) from the flu vaccine?** | Yes | | 76 | | 9.7% | 40 | | 52.6% | | 36 | | 47.4% | 16 | 21.1% | | 60 | | 78.9% | |
|  | No | | 710 | | 90.3% | 217 | | 30.6% | | 493 | | 69.4% | 66 | 9.3% | | 644 | | 90.7% | |
|  | ***P-value*** | | | | | ***<0.001*** | | | | | | | ***0.001*** | | | | | | |
| **Do you know anyone who has had moderate side effects (led to hospitalization) from the flu vaccine** | Yes | | 121 | | 15.4% | 59 | | 48.8% | | 62 | | 51.2% | 21 | 17.4% | | 100 | | 82.6% | |
|  | No | | 665 | | 84.6% | 198 | | 29.8% | | 467 | | 70.2% | 61 | 9.2% | | 604 | | 90.8% | |
|  | ***P-value*** | | | | | ***<0.001*** | | | | | | | ***0.007*** | | | | | | |
| **Do you know anyone who has had mild side effects from the flu vaccine?** | Yes | | 309 | | 39.3% | 144 | | 46.6% | | 165 | | 53.4% | 53 | 17.2% | | 256 | | 82.8% | |
|  | No | | 477 | | 60.7% | 113 | | 23.7% | | 364 | | 76.3% | 29 | 6.1% | | 448 | | 93.9% | |
|  | ***P-value*** | | | | | ***<0.001*** | | | | | | | ***<0.001*** | | | | | | |
| **Do you have** **enough information about the safety and side effects of the flu vaccine?** | Yes | | 289 | | 36.8% | 152 | | 52.6% | | 137 | | 47.4% | 59 | 20.4% | | 230 | | 79.6% | |
|  | No | | 497 | | 63.2% | 105 | | 21.1% | | 392 | | 78.9% | 23 | 4.6% | | 474 | | 95.4% | |
|  | ***P-value*** | | | | | ***<0.001*** | | | | | | | ***<0.001*** | | | | | | |
